# From genes to generations: genetic evaluation and counseling for infertility and pregnancy loss

**DOI:** 10.3389/fgene.2026.1764897

**Published:** 2026-06-08

**Authors:** Cristina Skrypnyk, Hussein Hifnawi AlHafnawi, Rawan AlHarmi, Essa Amin, Hafsa Albuarki

**Affiliations:** 1 Department of Molecular Medicine, AlJawhara Center, College of Medicine and Health Sciences, Arabian Gulf University, Manama, Bahrain; 2 Genetic Disease Clinic, University Hospital, King Abdullah Medical City, Manama, Bahrain; 3 Clinical Research Center, College of Medicine and Health Sciences, Arabian Gulf University, Manama, Bahrain; 4 Department of Urology, Ibn Al-Nafees Hospital, Manama, Bahrain; 5 Department of Obstetrics and Gynecology, King Hamad American Mission Hospital, A’ali, Bahrain

**Keywords:** chromosomal aberrations, early pregnancy loss, exome sequencing, genetic counseling, infertility, recurrent pregnancy loss

## Abstract

**Introduction:**

Understanding the genetics of reproductive disorders is key to improving diagnosis, treatment, and overall reproductive health. This study aimed to describe the characteristics, assessment, investigations, and recommendations in patients seeking genetic counseling for infertility and pregnancy loss.

**Methods:**

This is a retrospective cross-sectional study that included couples who presented to the Genetic Disease Clinic, University Hospital, King Abdullah Medical City, Bahrain, with a chief complaint of infertility or pregnancy loss over 13 years from 2012 to 2024.

**Results:**

Out of 912 patients who approached the clinic, 175 records (19.2%) belonged to couples (n = 350) who visited the clinic with infertility or pregnancy loss. Eighty couples (45.7%) were diagnosed with primary infertility, while 95 couples (54.3%) were diagnosed with secondary infertility. The mean marriage duration before visits was 7.7 ± 4.9 years. Meanwhile, 19 couples were identified as having advanced age in both partners (10.9%), 30 couples with advanced maternal age (17.1%), and 11 couples with advanced paternal age (6.3%). Additionally, couples with secondary infertility had an average of 1.6 pregnancies, with a mean of 1.4 early pregnancy losses. A male factor was identified in 30.3% of the couples (n = 53) and a combination of both male and female factors was reported in 52.0% (n = 91). Infertility and pregnancy loss were attributed to chromosomal aberrations solely in 27.4% of the couples (n = 48) and to monogenic carrier status in 14.3% of the couples (n = 25). Exome sequencing and gene panels were recommended and/or conducted in 49 couples (28%). *In vitro* fertilization and preimplantation genetic testing were recommended for couples with proven monogenic or chromosomal-related infertility.

**Conclusion:**

A couple’s genetic profile significantly impacts fertility potential and outcomes. Genetic counseling, screening, and diagnostic testing enable timely and personalized interventions.

## Introduction

Infertility is defined as the inability to conceive naturally. It is classified into two main types: primary infertility, where pregnancy has never been achieved, and secondary infertility, where pregnancy has been achieved at least once. It may stem from female factors, male factors, a combination of both or unexplained, when no identifiable cause is found (Geneva: World Health Organization, 2024). According to a report published by the World Health Organization (WHO) in 2023, the global lifetime prevalence of infertility is 17.5%. Moreover, the report indicated that the lowest lifetime prevalence of infertility, 10.7%, was reported from the Eastern Mediterranean region, where Bahrain is located (Geneva: World Health Organization, 2023). Furthermore, the global prevalence of couples who had two early pregnancy losses is 1.9%, and those who experienced it 3 times is 0.7% (Quenby et al., 2021).

Genetic infertility specifically refers to infertility attributed to genetic factors that impair reproductive functions. This includes chromosomal aberrations and single-gene disorders. Inherited conditions may impact reproductive tract anatomy, hormonal regulation, sperm production, ovulation or embryonic and fetal development. Genetic infertility may manifest in various ways, such as fertilization failure and recurrent pregnancy loss (RPL). Understanding the genetic basis of infertility is crucial for accurate diagnosis and effective personalized reproductive health management (Krausz and Riera-Escamilla, 2018; Yatsenko and Rajkovic, 2019).

Infertility and RPL are associated with psychological consequences, including stress, anxiety, and depression for both female and male partners, in addition to their economic burden (Quenby et al., 2021; Biggs et al., 2024; Inversetti et al., 2023; Nik Hazlina et al., 2022). Infertility and RPL are multifactorial health issues that could be related to chromosomal rearrangements and the genetic makeup in a percentage of cases (Poornima et al., 2020). Hence, the Centers for Disease Control and Prevention (CDC) listed infertility and early pregnancy loss among the indications for genetic counseling in addition to premarital and preconception counseling (CDC, 2024).

In view of the scarcity of published data and research regarding reproduction-related genetic consultations in the Gulf Cooperation Council (GCC) states in general and Bahrain in particular, this study sought to answer the following research question: what clinical characteristics, initial assessments, diagnostic investigations, and management recommendations are observed in patients seeking genetic assessment and counseling for infertility, pregnancy loss, and RPL? Accordingly, this study aimed to identify and describe these features in this patient population. The primary objective was descriptive, focusing on the clinical and diagnostic profiles of the cohort, while the secondary objective was analytical, assessing the relationship between patient-related variables and genetic findings and recommendations.

## Materials and methods

### Study population, setting, and design

This is a retrospective cross-sectional study that was conducted at the Genetic Disease Clinic, University Hospital, King Abdullah Medical City, Manama, Bahrain. The study included all couples who presented to the clinic with a chief complaint of infertility, pregnancy loss or RPL over the span of 13 years from 2012 to 2024. Data from the selected medical records were de-identified and collected in a Microsoft Excel sheet. Collected data included demographic data, medical and family history, referral type, initial assessment, previous investigations, genetic diagnostic testing, and management recommendations. Participants were categorized into primary and secondary infertility groups, as defined in the Introduction.

### Genetic evaluation and counseling sessions

In the initial genetic evaluation and counseling session, the patient’s data, family history, and previous investigations were reviewed to assess the possible cause and the need for further genetic testing. Each couple was informed about the proposed test, its purpose, procedures, result interpretation, accuracy, and limitations. Special attention was given to the emotional distress associated with a possible positive result, as well as to the couple’s right to make an autonomous decision to accept or decline the test. Where next-generation sequencing (NGS)-based testing was recommended, the possibility of secondary unexpected or incidental findings was communicated to guide their decision. Emphasis was also placed on confidentiality and the implications for the couples and their relatives.

**FIGURE 1:**
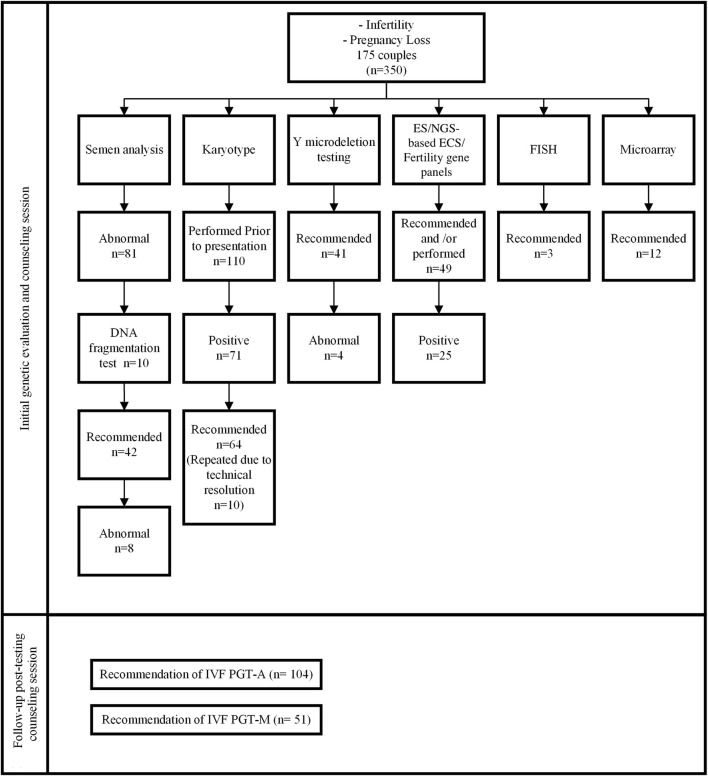
Flowchart of investigations performed for the study cohort. ECS: expanded carrier screening; ES, exome sequencing; FISH, fluorescence *in situ* hybridization; IVF, *in vitro* fertilization; NGS, next-generation sequencing; PGT-A, preimplantation genetic testing for aneuploidy; PGT-M, preimplantation genetic testing for monogenic disorders.

The follow-up post-testing counseling session aimed to facilitate patients’ understanding of their results and to support informed decision-making. Results were explained in terms of their implications and available management options. Emotional responses to the results, including feelings of anxiety or relief, were considered, and support was provided as needed. All appointments lasted at least 1 hour. Detailed reproductive planning options were discussed, covering choices such as preimplantation genetic testing for structural rearrangements (PGT-SR), aneuploidy (PGT-A), and monogenic disorders (PGT-M), as well as non-invasive and invasive prenatal testing and, when appropriate, natural conception (Figure 1).

### Statistical analysis

Statistical analysis was performed using the Statistical Package for the Social Sciences (SPSS®) software for Windows, version 28.0 (IBM, Armonk, NY, United States). Descriptive statistics were used to summarize the study sample’s demographics and medical history, while chi-square tables were used to identify the effects of various factors on the initial assessment and the subsequent recommendations. A significance level of probability (*p*) of less than 0.05 was considered statistically significant.

A structured, stepwise analytical approach was employed to evaluate predictors of the suspected etiology category (chromosomal, monogenic or multiple) and the recommended investigations and management options (karyotyping, Y microdeletion testing, *in vitro* fertilization (IVF) with PGT-A, and IVF with PGT-M). First, all candidate predictors were screened using bivariate analyses appropriate to variable type and distribution. Categorical predictors were assessed using chi-square or Fisher’s exact tests, with corresponding effect sizes (phi coefficient or Cramer’s V). Continuous predictors were tested for normality using the Shapiro–Wilk test; normally distributed variables were analyzed using independent-samples *t*-tests with Cohen’s *d*, while non-normally distributed variables (e.g., number of miscarriages) were analyzed using the Mann–Whitney U test with effect size *r*.

Predictors demonstrating statistical significance (*p* < 0.05), borderline significance (*p* < 0.1) or strong clinical relevance in bivariate analyses were considered for entry into multivariable logistic regression models, constructed separately for each outcome. Multivariable models were built using the enter method. Adjusted odds ratios (aORs) with 95% confidence intervals were reported. Model performance was evaluated using the omnibus test of model coefficients, Nagelkerke *R* ^2^, classification accuracy, and the Hosmer–Lemeshow goodness-of-fit test.

Restricting multivariable models to 5 predictors supported by bivariate evidence or strong clinical rationale minimized the risk of overfitting, sample size (n = 175), and missing data for some variables. Separate models were constructed for each recommendation to respect distinct clinical decision pathways, recognizing that recommended investigations (e.g., Y microdeletion testing) and therapeutic decisions (PGT-A and PGT-M) are governed by different mechanisms, and the possibility of recommending more than one. This approach allowed differentiation between predictors that are associated with an outcome in isolation and those that remain independently predictive after adjustment.

## Ethical approval

The study protocol was reviewed by the Research and Ethics Committee at the Arabian Gulf University, which granted it an exemption from full ethical review (reference number: E33-PI-02-24). The research was conducted in accordance with the ethical standards of the institution and with the Declaration of Helsinki. Informed consents were not required due to the retrospective design of the study.

## Results

### Population demographics and baseline characteristics

We reviewed 912 records of patients who approached the Genetic Disease Clinic from 2012 to 2024 with various complaints. Out of these, 175 records (19.2%) belonged to couples (n = 350 individuals) who visited the clinic with the chief complaint of pregnancy loss, RPL or infertility. Eighty couples (45.7%) were classified as having primary infertility, while 95 couples (54.3%) were classified as having secondary infertility. Our findings indicate that male partners were relatively older than female partners, as the mean age for males was 35.4 ± 6.1 years compared to 32.6 ± 5.8 years for females. We identified 19 couples (10.9%) with advanced age in both partners (males> 40 years, females> 35 years), only advanced maternal age in 30 couples (17.1%), and only advanced paternal age in 11 couples (6.3%). The data also show that couples visited the clinic at any point in the marriage, starting from 1 year after marriage till 22 years later. The mean marriage duration prior to clinic visits was 7.7 ± 4.9 years. Furthermore, couples with primary infertility had a mean marriage duration of 6.9 ± 4.4 (median = 6.0) years as opposed to couples with secondary infertility, with a mean of 8.7 ± 5.4 (median = 7.5) years. Although the data indicate that couples classified as having secondary infertility visited the clinic at a later point in their marriages, no statistical significance was found (*p* 0.1). Besides, the average number of pregnancies among couples with secondary infertility was 1.6, out of which a mean of 1.4 were early pregnancy losses (Table 1).

**TABLE 1:** Characteristics and reproductive history of the study cohort.

Risk factors and diagnostic tests (n = 175)	Primary infertility (n = 80) n (%)	Secondary infertility (n = 95) n (%)	Total count (%)
Age of male partner (n = 118)*	34.9 ± 5.8 (n = 54)	35.8 ± 6.3 (n = 64)	35.4 ± 6.1
Age of female partner (n = 149)*	32.3 ± 5.4 (n = 63)	32.8 ± 6.1 (n = 86)	32.6 ± 5.8
Marriage duration (n = 105)*	6.9 ± 4.4 (n = 59)	8.7 ± 5.4 (n = 46)	7.7 ± 4.9
IVF attempts	2.0 ± 2.4	1.1 ± 2.0	1.5 ± 2.2
Number of pregnancies	-	1.6 ± 2.0	-
Number of early pregnancy losses	-	1.4 ± 1.8	-
Number of live child/children	-	0.5 ± 0.7	-

Risk factors and diagnostic tests (n = 175)		Primary infertility (n = 80) n (%)	Secondary infertility (n = 95) n (%)	Total count (%)
Consanguinity	No	64 (48.9)	67 (51.1)	131 (74.9)
	Yes	16 (36.4)	28 (63.6)	44 (25.1)
Family history of infertility	No	74 (44.3)	93 (55.7)	167 (95.4)
	Yes	6 (75.0)	2 (25.0)	8 (4.6)
Family history of RPL	No	80 (48.5)	85 (51.5)	165 (94.3)
	Yes	0 (0)	10 (100.0)	10 (5.7)
Varicocele surgery	No	67 (42.9)	89 (57.1)	156 (89.1)
	Yes	13 (68.4)	6 (31.6)	19 (10.9)
Abnormal semen analysis	No	25 (26.9)	68 (73.1)	93 (53.1)
	Yes	55 (67.1)	27 (32.9)	82 (46.9)
Y microdeletion testing recommended	No	46 (34.3)	88 (65.7)	134 (76.6)
	Yes	34 (82.9)	7 (17.1)	41 (23.4)
Karyotype performed prior to presentation	No	29 (44.6)	36 (55.4)	65 (37.1)
	Yes	51 (46.4)	59 (53.6)	110 (62.9)
Positive karyotype findings (n = 110)	No	21 (53.8)	18 (46.2)	39 (35.5)
	Yes	30 (42.3)	41 (57.7)	71 (64.5)
Karyotype recommended	No	53 (47.7)	58 (52.3)	111 (63.4)
	Yes	27 (42.2)	37 (57.8)	64 (36.6)
IVF attempted prior to presentation	No	32 (36.4)	56 (63.6)	88 (50.3)
	Yes	48 (55.2)	39 (44.8)	87 (49.7)
IVF and PGT-A recommended	No	36 (50.7)	35 (49.3)	71 (40.6)
	Yes	44 (42.3)	60 (57.7)	104 (59.4)
IVF and PGT-M recommended	No	61 (49.2)	63 (50.8)	124 (70.9)
	Yes	19 (37.3)	32 (62.7)	51 (29.1)
Live child/children	No	80 (49.1)	63 (44.1)	143 (81.7)
	Yes	0 (0)	32 (100.0)	32 (18.3)
Infertility factors	Male	36 (67.9)	17 (32.1)	53 (30.3)
	Female	7 (36.8)	12 (63.2)	19 (10.9)
​	Both	35 (38.5)	56 (61.5)	91 (52.0)
	Unknown	2 (16.7)	10 (83.3)	12 (6.9)
Referral source	Obstetrics and Gynecology	60 (46.5)	69 (53.5)	129 (73.7)
	Andrology or Urology	16 (88.9)	2 (11.1)	18 (10.3)
	Others	1 (20.0)	4 (80.0)	5 (2.9)
	Self-referral	3 (13.0)	20 (87.0)	23 (13.1)
Infertility etiology	Anatomical	0 (0)	1 (100.0)	1 (0.6)
	Chromosomal	16 (33.3)	32 (66.7)	48 (27.4)
	Monogenic	8 (32.0)	17 (68.0)	25 (14.3)
	Undetermined	4 (40.0)	6 (60.0)	10 (5.7)
	Multiple	52 (57.1)	39 (42.9)	91 (52.0)

* Missing data; IVF: *in vitro* fertilization; n: sample size; PGT-A: preimplantation genetic testing for aneuploidy; PGT-M: preimplantation genetic testing for monogenic disorders; RPL: recurrent pregnancy loss; SD: standard deviation.

We found that a quarter of the cases reported consanguinity (n = 44, 25.1%), out of which 63.6% (n = 28) were classified as having secondary infertility. Additionally, only 8 out of 175 couples (4.6%) revealed a family history of infertility, of whom 6 couples (75.0%) were classified as having primary infertility. Moreover, 10 couples (5.7%) reported a family history of RPL, all within the secondary infertility group (Table 1). The study further shows that the majority of the couples (n = 152, 86.9%) were referred from other clinics, mainly obstetrics and gynecology (n = 129, 73.7%). Furthermore, 18 out of 175 couples (10.3%) were referred by an andrologist/urologist, out of which 16 (88.9%) were classified as having primary infertility, compared to two couples (11.1%) with secondary infertility (Table 1).

### Male factor infertility

Based on the previous investigations and medical reports available, infertility and pregnancy loss in 30.3% of the couples (n = 53) were attributed to male factors, with the majority being in the primary infertility group. Semen analysis reported a positive abnormal finding on the first visit in 46.9% of the male partners (n = 82), with a statistically significant association with primary infertility (*p* < 0.01) (Table 1). For the remaining couples, a semen analysis was recommended to confirm the presence of male-attributed infertility factor. Abnormalities identified included azoospermia, teratospermia, oligospermia, asthenozoospermia, and a combination of these sperm abnormalities. Among the 175 male partners who attended the clinic, only 10 (5.7%) had previously undergone sperm DNA fragmentation testing. DNA fragmentation testing was performed in a total of 52 patients (29.7%) and in 8 of them (15.4%), abnormal high fragmentation was reported, with values between 16% and 35%.

Azoospermia and other abnormalities in semen analysis were attributed to chromosomal aberrations in 29 cases (35.4%). These chromosomal aberrations were mostly polymorphisms, including 15ps+ (n = 6), 21ps+ (n = 5), 1qh+ (n = 3), and 22ps+ (n = 3), inversions (inv(2)(q31; q32.1)), translocations (t(13;14)(q10; q10)), and Y chromosomal abnormalities including Y microdeletion (Table 2). Other patients with sperm abnormalities also had *CFTR* gene variants (n = 6, 7.3%), in addition to two cases with Klinefelter syndrome (2.4%), of whom one was in a low mosaic state. Multivariable logistic regression was conducted for Y microdeletion testing recommendation following bivariate screening to avoid overfitting and collinearity (Supplementary Tables S1, S2). The final model demonstrated stable convergence, good calibration (Hosmer–Lemeshow *p* = 0.812), and meaningful explanatory power (Nagelkerke *R* ^2^ = 0.384). After adjustment for age, infertility history, and reproductive factors, positive semen analysis findings remained the only independent predictor (aOR 12.55, 95% CI 1.39–113.63; *p* = 0.024), male partners with abnormal semen analysis had 12.5-fold higher odds of Y microdeletion testing recommendation.

**TABLE 2:** Chromosomal aberrations in male partners with sperm abnormalities.

Chromosomal aberration	n
46,XY, 21ps+	4
46,XY, Y microdeletion	4
46,XY, large Y chromosome	4
46,XY, small Y chromosome	3
46,XY, 15ps+	3
46,XY, 16qh+	1
46,XY, 1qh+, 9qh+	1
46,XY, 1qh+, 16qh+	1
46,XY, inv (2)(q31; q32.1)	1
46,XY, 14ps+	1
46,XY, 13ps+, 15ps+, 22ps+	1
46,XY, 13ps+, 14 ps+, 21ps+	1
46,XY, 9qh-	1
45,XY, t (13; 14)(q10; q10)	1
46,XY, 15ps+, 22ps+	1
46,XY, 1qh+, 15ps+, 22ps+	1

In 19 couples (10.9%), male partners reported undergoing varicocele surgery, of which 13 (68.4%) were from the primary infertility group (Table 1). Our results reveal that a history of varicocele surgery is associated with positive abnormal findings in semen analysis (*p* < 0.01), as the majority of men who underwent varicocele surgery had sperm abnormalities (n = 18, 94.7%). Disorders of spermatogenesis identified in the study cohort included Sertoli cell-only syndrome (SCOS) in 5 patients and maturation arrest (MA) in two patients. Congenital absence of the vas deferens (CAVD) was present in two patients and congenital bilateral absence of vas deferens (CBAVD) was present in two patients as well. Other relevant factors and disorders reported included a history of cryptorchidism and/or orchidopexy (n = 3), sexual dysfunction (n = 1), testicular edema (n = 1), and previous inguinal hernia repair (n = 1).

### Female factor infertility

In the present study, infertility and pregnancy loss were attributed solely to female factors in 10.9% of the couples (n = 19), with the majority being in the secondary infertility group. Overall, diseases that affect reproduction in females that have been identified in the cohort included 10 women with thyroid disease (hypo- and hyperthyroidism), 8 women with polycystic ovary syndrome (PCOS), 7 women with uterine factor infertility, 6 with other hormonal imbalances, 4 with other ovarian factor infertility, 3 with thrombotic disorders, two with tubal factor infertility, two with cervical factor infertility, one patient with collagenopathy, and one patient with both systemic lupus erythematosus (SLE) and sickle cell disease (SCD).

Multiple factors were postulated to contribute to infertility or pregnancy loss in 91 couples (52.0%) and in 10 couples (5.7%), the etiology was undetermined. Among the possible factors that might contribute to infertility in both males and females, we identified 13 patients with obesity (3.7%), of whom two underwent a sleeve gastrectomy previously. Diabetes mellitus was identified in two patients (0.6%). Autoimmune diseases were identified in 4 patients (1.1%). Moreover, infertility and pregnancy loss were suspected to result from other factors such as smoking, environmental and workplace exposures, and viral diseases. Additionally, Potter syndrome was suspected in the lost pregnancies of 3 couples.

### Genetic findings

#### Chromosomal aberrations

Karyotyping was carried out in 110 couples (62.9%), of these, 71 (64.5%) reported chromosomal infertility-related findings. It was repeated in 10 couples due to technical or resolution issues. Chromosomal aberrations were suggested to account for infertility and pregnancy loss in a total of 42.9% of the couples (n = 75), including couples where other factors were also identified. The aberrations included chromosomal rearrangements (inversions, translocations or duplications) and polymorphisms. Chromosomal polymorphisms were the most common abberation identified and were reported in both partners in 13 couples, only in female partners in 8 couples, and only in male partners in 32 couples. In our cohort, the most common polymorphisms in females were 1qh+ and 9qh+, and 21ps+ in males. Our results also showed that 25 partners of those with karyotype findings had a variation in chromosome 9. Additionally, 6 patients had a large Y chromosome and 3 had a small Y chromosome.

**FIGURE 2:**
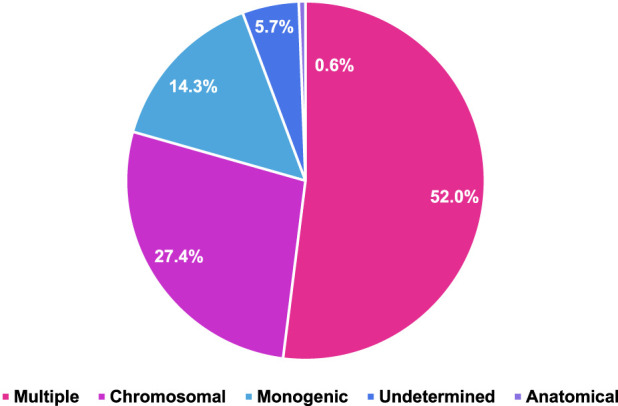
Root causes of infertility based on the assessment of the study cohort. Pie chart displaying five categories: Multiple causes at 52.0 percent, Chromosomal at 27.4 percent, Monogenic at 14.3 percent, Undetermined at 5.7 percent, and Anatomical at 0.6 percent.

Other chromosomal aberrations were identified, including mosaic Turner syndrome 46,XX/45,X (n = 3), mosaic triple X syndrome 47,XXX/46,XX (n = 1), Klinefelter syndrome 47,XXY (n = 1), low mosaic Klinefelter syndrome 46,XY [23]/47,XXY [2] (n = 1), and a marker chromosome 47,XY,+mar (n = 2). Y microdeletion testing was recommended for 41 males (23.4%), of whom 82.9% (n = 34) were classified as having primary infertility. Among those who underwent testing, 39 patients had sperm abnormalities (95.1%) and 7 patients with a history of varicocele and/or varicocele surgery (17.1%). Four patients (9.8%) had a positive test result. All patients with a Y microdeletion had azoospermia. Microarray testing was recommended for 12 patients and fluorescence *in situ* hybridization (FISH) was recommended for 3 patients (Tables 1, 3, 4; Figure 2). Additionally, one patient presented with a germ cell division defect and another one with a mitotic spindle defect.

**TABLE 3:** Chromosomal aberrations identified in the study cohort.

Chromosome	Aberration	Male partner (n)	Female partner (n)
**1**	qh+	5	5
	t (1; 12)(q43; q24)	0	1
	t (1; 14)(p36.33; qter)	0	1
**2**	inv (2)(q31; q32.1)	1	0
**9**	qh+	8	5
	qh-	2	1
	t (9; 22)(q13; p11.2)	0	1
	t (9; 11)(q33-qter; 11q25-qter)	0	1
	inv (9)(p12; q13)	0	3
	inv (9)(p13; q21)	1	0
	inv (9)(p11; q11)	0	1
	inv (9)(p11; q12)	1	0
	inv (9)(p11; q13)	0	1
**13**	ps+	2	1
	t (13; 14)(q10; q10)	0	1
**14**	ps+	3	1
**15**	ps+	8	5
**16**	qh+	4	2
**21**	ps+	8	3
**22**	ps+	7	1
**?**	mark	2	0
**Y**	microdeletion	4	-
	large size	6	-
	small size	3	-
Sex chromosome aneuploidies		n	
47,XXY		1	
46,XX/45,X		3	
46,XX/47,XXX		1	
46,XY/47,XXY		1	

**TABLE 4:** Frequency of chromosomal polymorphisms in the study cohort.

Polymorphisms	n
One chromosomal polymorphic variation per couple	31
Two chromosomal polymorphic variations per couple	11
Three chromosomal polymorphic variations per couple	2
Four chromosomal polymorphic variations per couple	3
Polymorphisms in male partner only	32
Polymorphisms in female partner only	8
Polymorphisms in both partners	13

For karyotype recommendation, both bivariate and multivariable analyses consistently showed no meaningful associations with demographic, clinical or reproductive predictors (Supplementary Tables S3, S4). Multivariable logistic regression confirmed the absence of independent predictors, with all aORs close to unity and small or negligible effect sizes. The model showed stable convergence, good calibration (Hosmer–Lemeshow *p* = 0.911) but low explanatory power (Nagelkerke *R* ^2^ = 0.119). Karyotype recommendation in this cohort was not systematically influenced by patient-level characteristics.

#### Monogenic disorders

Among the 175 couples who visited the clinic, only 6 (3.4%) presented initially with genetic testing reports that could explain infertility or pregnancy loss. Exome sequencing (ES) and/or NGS-based preconception expanded carrier screening (ECS) and fertility gene panels were recommended and/or performed in 49 couples (28%). In 14.3% of the couples (n = 25), infertility or pregnancy loss could be explained solely by a rare autosomal recessive (AR) carrier status or possible germinal mosaicism of a rare dominant *de novo* mutation (Table 1; Figure 2). Disorders identified in the cohort, whether in the heterozygous or homozygous state, included cystic fibrosis (*CFTR*) (n = 6), Joubert syndrome 6 (*TMEM67*) (n = 1) and 11 (*TTC21B*) (n = 2), mitochondrial DNA depletion syndrome 6 (*MPV17*) and Chédiak-Higashi syndrome (*LYST*) (n = 2), phenylketonuria (*PAH*) (n = 2), Zellweger syndrome (*PEX1*) (n = 2), Sandhoff disease (*HEXB*) (n = 2), Niemann-Pick disease (*SMPD1*) (n = 2), fragile X syndrome (*FMR1*) (n = 2), primary ovarian insufficiency (POI) 13 (*MSH5*) (n = 1), spermatogenic failure (SPGF) 5 (*AURKC*) (n = 1), SPGF 11 (*KLHL10*) (n = 1), SPGF 12 (*NANOS1*) (n = 1), SPGF 34 (*FSIP2*) (n = 1), SPGF 39 (*DNAH17*) (n = 2), SPGF 53 (*ACTL9*) (n = 1), SPGF 56 (*DNAH10*) (n = 1), SPGF, X-linked 3 (*CFAP47*) (n = 1), primary ciliary dyskinesia 3 (*DNAH5*) (n = 1), acute intermittent porphyria (*HMBS*) (n = 1), Wilson disease (*ATP7B*) (n = 1), Dubin-Johnson syndrome (*ABCC2*) (n = 1), CBAVD, X-linked (*ADGRG2*) (n = 1), and Familial Mediterranean Fever (FMF) (*MEFV*) (n = 1) (Table 5). Other disorders identified in the cohort included hemoglobinopathies (SCD, thalassemias, and sickle cell-hemoglobin D disease) and G6PD deficiency.

**TABLE 5:** Monogenic disorders identified in the study cohort.

Disorder	Gene	Inheritance	n	Variant(s)	Protein change	Classification	ClinVar ID	rsID	Allele frequency	CADD score	Phenotype	Reported association
CF	*CFTR*	AR	6	c.2989-1G>A	-	P	53,613	rs397508470	6.2 × 10^−7^	34.0	Azoospermia, obesity, positive family history of CF, CAVD, CBAVD	Almost all of these variants are associated with infertility and CBAVD (Landrum et al., 2014; Gaikwad et al., 2018; Yoon et al., 2019)
				c.3909C>G	p.Asn1303Lys	P	7,136	rs80034486	1.6 × 10^−4^	23.5		
				c.1411A>T	p.Ile471Phe	LP/VOUS	2150527	rs1459732115	1.2 × 10^−6^	25.1		
				c.389T>C	p.Leu130Pro	VOUS	1029874	rs1798853976	Not found	28.0		
				c.1408G>A	p.Val470Met	B/LB	7,130	rs213950	0.4	14.6		
Joubert syndrome 6	*TMEM67*	AR	1	c.1054G>A	p.Gly352Ser	LP	Not reported	rs2536847115	6.2 × 10^−7^	24.3	RPL	Possible fetal outcomes (Strong et al., 2021)
Joubert syndrome 11	*TTC21B*	AD, AR	2	c.2703A>T	p.Glu901Asp	B	Not reported	rs1355515906	Not found	16.0		
Phenylketonuria	*PAH*	AR	2	c.1184C>G	p.Ala395Gly	P/LP	102,549	rs62508736	6.2 × 10^−6^	31.0	RPL	RPL might be attributed to variant co-occurrence
MDDS	*MPV17*	AR	2	c.278A>C	p.Gln93Pro	P/LP	694,362	rs200938111	6.2 × 10^−7^	33.0	RPL in addition to two children who died in infancy; one with CHS and the 2nd with MDDS	RPL might be attributed to variant co-occurrence
CHS	*LYST*	AR	2	c.2311C>T	p.Gln771Ter	LP	Not reported	Not reported	Not found	36.0		
				c.146G>A	p.Gly49Asp	VOUS	959,277	rs2527300575	1.2 × 10^−6^	25.0		
ZS	*PEX1*	AR	2	c.730del	p.Ser244ValfsTer5	VOUS	Not reported	Not reported	Not found	Not found	RPL with suspected ZS	Pregnancy loss (Johnson et al., 2001)
Sandhoff disease	*HEXB*	AR	2	c.1238_1242del	p.Lys414CysfsTer7	P	93,196	rs398123445	Not found	Not found	RPL in addition to one child who died in infancy	Pregnancy loss (Tavasoli et al., 2018)
NPD	*SMPD1*	AR	2	c.1522G>A	p.Gly508Arg	B	93,317	rs1050239	0.2	19.3	RPL with NPD	Pregnancy loss (Hellani et al., 2004)
FXS	*FMR1*	XLD	2	CGG 55 premutation	-	P	9,972	Not reported	Not found	Not applicable	POI, lost one child with a cardiac defect, another patient with an autistic child that also has FXS	Infertility and POI risk (Allen et al., 2021)
				CGG 87 premutation	-	P	9,972	Not reported	Not found	Not applicable		
POI 13	*MSH5*	AR	1	c.1051C>G	p.Arg351Gly	VOUS/B	1256029	rs28399976	0.02	24.6	Primary infertility	Infertility (Yatsenko and Rajkovic, 2019)
CBAVD, XL	*ADGRG2*	XL	1	c.521T>A	p.Ile174Lys	VOUS	Not reported	Not reported	Not found	20.0	Primary infertility	Infertility (Cai and Li, 2022)
SPGF 5	*AURKC*	AR	1	c.762A>G	p.Val254 =	VOUS	893,271	rs55898757	1.5 × 10^−4^	3.1	Primary infertility	Infertility (Ortega et al., 2020)
SPGF 11	*KLHL10*	AD	1	c.1014A>C	p.Lys338Asn	VOUS	2433163	rs2544489279	Not found	15.0	Oligospermia, asthenozoospermia, teratospermia	Infertility (Guzmán-Jiménez et al., 2024)
SPGF 12	*NANOS1*	AD	1	c.458C>G	p.Pro153Arg	LB	Not reported	Not reported	8.0 × 10^−7^	23.6	Oligoteratozoo-spermia	Infertility (Kusz-Zamelczyk et al., 2013)
SPGF 34	*FSIP2*	AR	1	c.11594G>C	p.Ser3865Thr	B	Not reported	rs866217971	3.2 × 10^−6^	4.8	Primary infertility	Infertility (Guzmán-Jiménez et al., 2024)
				c.163G>A	p.Val55Ile	B	Not reported	rs867177922	5.9 × 10^−6^	19.0		
SPGF 39	*DNAH17*	AR	2	c.4504A>T	p.Ile1502Phe	LP	Not reported	rs538711781	3.2 × 10^−5^	27.5	Primary infertility	Infertility (Whitfield et al., 2019)
				c.4473G>C	p.Glu1491Asp	VOUS	Not reported	rs2090522590	Not found	17.0		
				c.3844C>T	p.Leu1282Phe	VOUS	Not reported	rs201391989	2.4 × 10^−5^	25.4		
				c.5955_5956delCAinsGG	p.Met1986Val	VOUS	Not reported	rs34426129	Not found	Not found		
SPGF 53	*ACTL9*	AR	1	Report is unavailable	-	-	-	-	-	-	Primary infertility	Infertility (Guzmán-Jiménez et al., 2024)
SPGF 56	*DNAH10*	AR	1	Report is unavailable	-	-	-	-	-	-	Oligoastheno-teratozoospermia	Infertility (Tu et al., 2021)
SPGF, XL 3	*CFAP47*	XLR	1	c.10C>T	p.Gln4Ter	LB	3054256	rs141633156	3.5 × 10^−4^	25.0	Primary infertility, multiple IVF failures	Infertility (Liu et al., 2023)
Primary ciliary dyskinesia 3	*DNAH5*	AR	1	Report is unavailable	-	-	-	-	-	-	Azoospermia	Infertility (He et al., 2020)
Acute intermittent porphyria	*HMBS*	AD	1	Report is unavailable	-	-	-	-	-	-	Primary infertility, abnormal semen analysis	Not reported in the literature to cause infertility; this may be attributed to variant co-occurrence and other factors
Wilson disease	*ATP7B*	AR	1	Report is unavailable	-	-	-	-	-	-	RPL	The male partner is affected in this couple but subfertility and pregnancy loss have been reported (Malik et al., 2013)
Dubin-Johnson syndrome	*ABCC2*	AR	1	Report is unavailable	-	-	-	-	-	-	Primary infertility, abnormal semen analysis	Not reported in the literature to cause infertility; this may be attributed to variant co-occurrence and other factors
Familial Mediterranean Fever	*MEFV*	AD, AR	1	c.605G>A	p.Arg202Gln	B	36,512	rs224222	0.2	0.001	RPL	Infertility and RPL (Atas et al., 2020; Yasar et al., 2014)
				c.2080A>G	p.Met694Val	P/LP	2,538	rs61752717	1.8 × 10^−4^	9.0		

Mode of inheritance is based on Online Mendelian Inheritance in Man (OMIM) data (McKusick-Nathans Institute of Genetic Medicine, 2024). Variant annotations, including variant, protein change, classification, and ClinVar ID, were derived from ClinVar (Landrum et al., 2014). Variant classification and protein change were also supported by patient reports, VarSome, and GeneBe (Kopanos et al., 2019; Stawiński and Płoski, 2024). rsIDs, were retrieved from dbSNP (Sherry et al., 2001). Allele frequencies and *in silico* predictions were obtained from gnomAD, and supported by GeneBe (Stawiński and Płoski, 2024; Karczewski et al., 2020). AD: autosomal dominant; AR: autosomal recessive; B: benign; CADD: combined annotation dependent depletion; CAVD: congenital absence of the vas deferens; CBAVD: congenital bilateral absence of the vas deferens; CF: cystic fibrosis; CHS: Chédiak-Higashi syndrome; FXS: fragile X syndrome; IVF: *in vitro* fertilization; LB: likely benign; LP: likely pathogenic; MDDS: mitochondrial DNA, depletion syndrome 6; NPD: Niemann-Pick disease; P: pathogenic; POI: primary ovarian insufficiency; RPL: recurrent pregnancy loss; SPGF: spermatogenic failure; VOUS: variant of uncertain significance; XL: X-linked; XLD: X-linked dominant; XLR: X-linked recessive; ZS: Zellweger syndrome.

Initial univariate screening identified several categorical and continuous predictors associated with etiological classification (Supplementary Tables S5–S7). Given instability in multinomial models, etiologies subsequently collapsed into “single” versus “multiple” categories, and a multivariable binary logistic regression was performed. In the adjusted model, a positive karyotype finding was independently associated with etiology, with significantly lower odds of the multiple etiology category (aOR 0.29, 95% CI 0.10–0.87; *p* = 0.027), corresponding to a moderate effect size (Cohen’s *d* = −0.67), whereas female age, consanguinity, semen analysis findings, and infertility type showed small or negligible effects (Supplementary Table S8). This stabilized model converged appropriately and demonstrated acceptable calibration (Hosmer–Lemeshow *p* = 0.066), with modest explanatory power (Nagelkerke *R* ^2^ = 0.126).

### Clinical recommendations

In the initial assessment, 87 couples (49.7%) reported having undergone IVF at least once. Of these, 39 (44.8%) confirmed having achieved at least one prior pregnancy. Of the couples who reported undergoing IVF previously, 60 couples (69.0%) underwent 1-3 trials, 19 couples (21.8%) underwent 4-6 trials, and 8 couples (9.2%) underwent 7-12 trials. Sixty-one couples (70.1%) underwent more than one IVF trial, out of which 50 couples (83.3%) have had karyotyping done and 32 couples (64.0%) reported a positive karyotype finding. Out of the couples who already underwent 7-12 IVF trials, only 3 of them (37.5%) presented to our clinic with a positive karyotype finding. Following the initial assessment, we recommended IVF and PGT-A for 104 couples (59.4%) and IVF with PGT-M for 51 couples (29.1%), mainly for those diagnosed with secondary infertility (Table 1).

For IVF with PGT-A recommendation, bivariate analyses showed significant associations with female age, infertility etiology, and positive karyotype findings (Supplementary Tables S9, S10). Multivariable logistic regression model confirmed the independent effects of all 3 factors. Each 1-year increase in female age increased the odds of PGT-A recommendation (aOR 1.62, 95% CI 1.04–2.54; *p* = 0.034), while positive karyotype findings emerged as the strongest predictor (aOR 342.71, 95% CI 5.78–20,320.37; *p* = 0.005), with large effect sizes. Infertility etiology (female) also remained independently associated (overall *p* = 0.014). These results demonstrate a clear linkage between the predictors and the outcome after adjustment for confounders. The model showed stable convergence, excellent calibration (Hosmer–Lemeshow *p* = 0.545), and high explanatory power (Nagelkerke *R* ^2^ = 0.726).

For IVF with PGT-M recommendation, multivariable logistic regression was used to account for overlapping reproductive and genetic risk factors identified in bivariate analyses (Supplementary Tables S11, S12). Although consanguinity, positive history of diseases affecting reproduction in females, and marriage duration showed significant or borderline associations in univariate analyses, none retained independent statistical significance after adjustment, and effect sizes were small. Model calibration was excellent (Hosmer–Lemeshow *p* = 0.997), but explanatory power was modest (Nagelkerke *R* ^2^ = 0.223), indicating that PGT-M recommendation reflects multifactorial clinical judgment rather than reliance on a single determinant.

## Discussion

Infertility is heavily influenced by the genetic landscape in both males and females. The genetic causes of infertility are diverse and complex. In the present study, we report 175 couples who presented to the Genetic Disease Clinic with infertility, early pregnancy loss, and RPL. The approach to infertile couples is initiated by andrological and gynecological examination, imaging, and hormonal assessment, followed by the appropriate investigations depending on the clinical suspicion, including semen analysis, karyotyping, and genetic testing (specific genes and panels) (Wyrwoll et al., 2021).

Chromosomal aberrations are an established cause of infertility and RPL. Chromosomal rearrangements, deletions, inversions, and translocations interfere with normal gamete formation and embryonic loss or fetal death, leading to infertility (Krausz and Riera-Escamilla, 2018; Yatsenko and Rajkovic, 2019). We found chromosomal aberrations in 42.9% of the cases, including those with other contributing etiologies, with chromosomal polymorphisms and rearrangements being the most common findings, possibly associated with an increased risk for abnormal chromosomal segregation and abnormal products of conception. Karyotype analysis revealed chromosomal abnormalities in 64.5% of the couples analyzed. In contrast to other studies, our cohort exhibited a higher prevalence of chromosomal abnormalities, including neighboring countries (Poornima et al., 2020; Clementini et al., 2005; Dutta et al., 2011; Pal et al., 2018; Beg et al., 2019; El-Dahtory et al., 2022). Globally, prevalence rates of chromosomal abnormalities among infertile males range from 2% to 13.5% and from 0.5% to 2.5% in infertile females, including data from research conducted in some Arab countries (Clementini et al., 2005; Vicdan et al., 2004; Mierla et al., 2015; Elghezal et al., 2006; Yatsenko et al., 2010; Kayed et al., 2006).

Chromosomal polymorphisms, variants in the heterochromatic, non-coding regions of DNA repeats, have been regarded as benign, normal and harmless variations in older studies. However, their incidence seems to be higher in the infertile population, including studies with fertile controls, suggesting an association (Ralapanawe et al., 2023; Hsu et al., 1987; Zhao et al., 2022; Minocherhomji et al., 2009). Heterochromatin has been implicated in maintaining genomic stability and segregation of chromosomes. It has been postulated that chromosomal polymorphisms may elevate the risk of meiotic errors, impact oocyte maturation, and contribute to aneuploidy (Lu et al., 2024). Some studies support polymorphisms affecting meiotic pairing, segregation, and gene regulation through position-effect variegation (Pires et al., 2024). In our cohort, the most common polymorphisms in females were 1qh+ and 9qh+, and 21ps+ in males. Chromosome 9 possesses the highest degree of variation among non-acrocentric chromosomes (Kosyakova et al., 2013). For instance, our results show that 25 partners of those with karyotype findings had a variation in chromosome 9. Seven partners were identified to have pericentric inversions of chromosome 9 (2%) (Table 3) which are considered balanced structural aberrations that have an overall frequency of 1.5% in the general population and 30% in couples with RPL (Kosyakova et al., 2013; Kumar et al., 2012). Studies have reported that carriers of such balanced structural aberrations are at an increased risk of having offspring with an unbalanced karyotype. The probability of them producing abnormal gametes as a result of meiotic crossover can be up to 30%. One potential approach to mitigate this risk is by employing IVF and preimplantation genetic testing (PGT) (Minocherhomji et al., 2009; Purandare et al., 2011). Furthermore, in males with azoospermia and semen analysis abnormalities, the most common polymorphisms were 15ps+ (n = 6), 21ps+ (n = 5), 1qh+ (n = 3), and 22ps+ (n = 3). Genes responsible for asthenozoospermia are located on chromosomes 9 (*DNAI1*) and 15 (*DYX1C1*). Additionally, genes implicated in non-obstructive azoospermia (NOA) include *DMRT1*, *SOHLH1*, and *NR5A1* are also located on chromosome 9 (Kuroda et al., 2020). However, as mentioned earlier, evidence remains conflicting as other studies have reported that polymorphisms are unlikely to result in a change in phenotype given they occur in non-coding regions (Ralapanawe et al., 2023; Mierla and Stoian, 2012). Moreover, Robertsonian translocations occur in acrocentric chromosomes, including chromosomes 13, 14, 15, and 21, resulting in sperm aneuploidy, which can lead to miscarriage or trisomy (Kuroda et al., 2020). Interestingly, a couple has a child with intellectual disability and microduplication 1q21.2, with no family history or parental microduplication, suggesting a *de novo* event during gamete formation. Since array testing cannot rule out a balanced chromosomal rearrangement, there remains a recurrence risk for future pregnancies although it is low. However, this risk may potentially contribute to RPL and IVF failures.

Large Y chromosome, designated as Yqh+, refers to a morphologically enlarged or long Y chromosome, attributed to the expansion of heterochromatic non-coding material on its long arm. It has been long considered a benign variant (Nie and Lu, 2011). However, it has been noted to be common in infertile men and to possibly affect IVF outcomes (Videaú et al., 2001; Xiao et al., 2012). Small Y chromosome (Yqh-), on the other hand, denotes a morphologically small Y chromosome due to reduced length or absence of heterochromatin on the long arm of the chromosome. Like Yqh+, it is considered a normal variant. However, it has been observed to be significantly frequent in azoospermic patients (Li et al., 2015). These patients are also more likely to harbor AZF microdeletion (Fu et al., 2023).

Male infertility can stem from several genetic factors. Y chromosome microdeletion is a significant cause of male infertility, resulting in oligospermia and azoospermia. Moreover, conditions like Klinefelter syndrome (47,XXY) and other sex chromosome abnormalities contribute to male infertility by impairing spermatogenesis (Kuroda et al., 2020). Additionally, pathogenic variants in the *CFTR* gene, which is associated with cystic fibrosis, contribute to oligospermia and several obstructive reproductive anomalies in males, leading to infertility (Bieniek et al., 2021). In the present study, 23.4% of the male partners were recommended to undergo Y microdeletion testing, with most cases associated with primary infertility. Azoospermia and other sperm abnormalities were attributed to chromosomal abnormalities in 29 cases, of which 4 cases had Y microdeletion, in addition to two cases with Klinefelter syndrome, of whom one was in a low mosaic state. *CFTR* gene variants were reported in 6 of these cases, highlighting the importance of *CFTR* testing in male infertility. Almost all detected *CFTR* variants were associated with CBAVD (Landrum et al., 2014; Gaikwad et al., 2018).

In females, chromosomal abnormalities such as Turner syndrome (45,X) and X-autosome translocations, along with genomic variants linked to conditions such as PCOS, are common contributors to infertility (Yatsenko and Rajkovic, 2019). In our study, 3 mosaic Turner syndrome cases were identified, underlying the importance of consideration of this pathology during female infertility assessment. Also, 12 female partners (6.9%) were affected by ovarian diseases (including PCOS and POI), in line with literature that highlights the genetic predisposition associated with these conditions (Yatsenko and Rajkovic, 2019). Two individuals with secondary infertility were identified as carriers of fragile X syndrome, each heterozygous for an *FMR1* premutation, with one exhibiting 55 CGG repeats and the other 87 CGG repeats. The latter is a mother of a daughter affected by autism and confirmed by testing as having a full mutation *FMR1* allele, inherited from her mom. Findings by Allen et al. (Allen et al., 2021) reveal that women with 85–89 repeats have the highest risk for POI. On the other hand, women with 55–64 repeats did not show an elevated risk. Interestingly, high-normal alleles ranging from 35-54 repeats have been shown to be associated with secondary infertility in another study and generally conflicting results (Allen et al., 2021; Grasmane et al., 2021). Additionally, one patient had SLE and SCD, both of which affect fertility (Stamm et al., 2022; Pecker and Cameron, 2024). Pertaining to SCD and contrary to popular belief, a retrospective study showed pregnancy loss in patients with sickle cell trait (Taylor et al., 2008). Other diseases that could contribute to early pregnancy loss and RPL that were tested and identified in our cohort included thrombotic, autoimmune, and thyroid diseases, which could impact the ovarian function through systemic inflammation and hormone dysregulation (Popa et al., 2025). These observations should be interpreted within a couple-based framework, recognizing that reproductive outcomes reflect the combined genetic contributions of both partners, particularly in consanguineous populations.

Consanguinity elevates the risk of genetic disorders, such as AR disorders, which can contribute to infertility (Tadmouri et al., 2009). The reported prevalence of consanguineous marriages ranges from 29.7% to 54% in the GCC, higher than regions such as the Americas, Europe, and Australia (Temaj et al., 2022). We report consanguinity in 25.1% of the analyzed cases, with a higher occurrence among couples experiencing secondary infertility (63.6%). A study in Lebanon reported the presence of family clustering of male factor infertility (Inhorn et al., 2009). A study conducted in Kuwait reported an association between parental consanguinity and reduced ovarian reserve, postulating a correlation with AR genes (Seher et al., 2015). AR disorders, such as cystic fibrosis and various metabolic disorders, are more likely to be inherited by the offspring of consanguineous couples, often resulting in pregnancy loss and affecting fertility. The increased incidence of these disorders in consanguineous couples reflects the higher likelihood of shared genetic material, which amplifies the expression of deleterious alleles. In regions with high rates of consanguinity, genetic counseling is crucial for reproductive health, as it helps identify at-risk couples for AR disorders and provides guidance on genetic testing and family planning options (Oniya et al., 2019). It is worth noting that miscarriages can be the consequence of a lethal gene variant while the pedigree is not suggestive of any genetic disorder and there is no consanguinity.

Genetic counseling, as well as premarital and preconception counseling, are of paramount importance in managing the reproductive health of consanguineous, as well as non-consanguineous couples. A total of 19.2% of the visits to our clinic were related to infertility and pregnancy loss. The average number of pregnancies among couples with secondary infertility was 1.6, out of which a mean of 1.4 were early pregnancy losses. Preconception ECS and PGT can be recommended accordingly to identify couples at genetic risk before conception or during early embryo development. To reduce pregnancy loss attributed to structural chromosomal abnormalities, PGT-SR can be utilized, where a balanced, euploid embryo is used for transfer (Sierra et al., 2024). These strategies reduce the chances of infertility and pregnancy loss and are especially valuable in high-consanguinity regions like Bahrain. In contrast, other factors contribute to infertility in other parts of the world, such as environmental factors in industrialized regions and secondary infertility being common in areas with a high prevalence of sexually transmitted infections, each of which can be addressed differently (Skakkebæk et al., 2022; Fauser et al., 2024). A multidisciplinary approach encompassing genetic counseling, diagnostic testing, and assisted reproductive technologies (ART) is vital, especially in cases involving chromosomal abnormalities, to facilitate the selection of healthy embryos for implantation. Our study highlights the significance of genetic counseling, as 59.4% of the couples received recommendations for IVF and PGT-A and 29.1% for PGT-M which can help in avoiding recurrence risks. Based on our analysis, we found that PGT-M recommendation reflects multifactorial clinical judgment rather than reliance on a single determinant. Noteworthily, an IVF with a failed outcome should be followed by detailed investigations prior to the next trial. In the present study, among the 61 couples (70.1%) who underwent multiple IVF trials, 50 (83.3%) had karyotyping performed, with 32 (64.0%) showing positive karyotype results. Among those who underwent 7-12 trials, 3 couples (37.5%) had positive karyotype findings. Identifying a genetic cause promptly in such cases can potentially result in a better outcome. Moreover, we recommended ECS in couples where all of the investigations did not identify a possible genetic cause to explain RPL, especially in consanguineous couples. In such cases, infertility could be explained by rare AR carrier status or possible germinal mosaicism of a rare dominant *de novo* mutation.

Delays in diagnosing and treating infertility can diminish the likelihood of success of interventions such as IVF (Shingshetty et al., 2024). Couples who postpone seeking help for infertility often experience increased emotional distress (Domar et al., 2021). Bereavement might contribute to late consultation and diagnosis. The couples in our study have been married for a mean of 7.7 ± 4.9 years at the time of the visit. Couples classified as having secondary infertility visited the clinic at a later point in their marriages, however the association was not statistically significant. Similar studies from other GCC countries report comparable delays in diagnosis and treatment (Al-Turki, 2015). We also identified 19 couples with advanced age in both partners (males> 40 years, females> 35 years), only advanced maternal age in 30 couples, and only advanced paternal age in 11 couples (Malamitsi‐Puchner and Briana, 2023). Delaying seeking help can result in reaching an advanced biological age, where the risks of aneuploidies and RPL are already elevated. Advanced paternal age has been linked to decreased sperm quality and testicular function, genetic abnormalities, and epigenetic modifications (Kaltsas et al., 2023). Advanced maternal age, on the other hand, affects oocyte quality and leads to ovarian reserve decline (Chu et al., 2023). Some couples were beyond the window of conception at the time of presentation to the clinic but were just pursuing a state of inner peace and reassurance to understand if indeed an intervention could have been done earlier. After proper counseling and a thorough obstetrical evaluation, genetic testing may not be deemed useful in such cases as it would not provide any benefits to the couple. This delay in seeking medical advice can be multifactorial, with an interplay of social and cultural stigma, lack of knowledge and awareness, accessibility, and personal factors, often leading to delayed infertility diagnosis and management (Domar et al., 2021; Abolfotouh et al., 2013). In cases of secondary infertility, couples may delay treatment assuming that a previous successful pregnancy guarantees continued fertility.

### Study limitations

Limitations of this study include the retrospective design and the sample size, with the majority of patients referred by other specialties, which is not representative of the general population. It is also important to note that certain data are missing, which may affect the comprehensiveness of the findings. Moreover, many patients lost follow-up, resulting in a lack of records of the results of the tests that were recommended to them. The study does not address mitochondrial disorders and their impact on reproduction as none of the patients had a suggestive picture to be tested accordingly. Finally, in the absence of a matched control group (e.g., fertile couples), the interpretation of our findings is limited, as we are unable to determine the true prevalence or significance of the identified genetic variants relative to the general population. Therefore, our results should be interpreted as observations within a referred population rather than evidence of a definitive association. More future studies with fertile controls are necessary to establish whether these findings represent true associations or reflect normal variation.

## Conclusion

In essence, our findings from this retrospective study suggest that the genetic makeup of a couple is associated with shaping their fertility potential and outcomes. Of patients visiting the Genetic Disease Clinic, 19.2% were couples who reported infertility or pregnancy loss, a predominantly referral-based cohort. Among them, 45.7% (80 couples) had primary infertility. Both partners appeared to contribute to infertility and pregnancy loss in 91 couples (52.0%). Infertility and pregnancy loss were solely attributed to chromosomal aberrations in 48 couples (27.4%) and monogenic carrier status in 25 couples (14.3%). Those with secondary infertility averaged 1.6 pregnancies and 1.4 early losses. Identifying these genetic factors through genetic counseling, followed by screening and diagnostic genetic testing, is essential for employing early and appropriate interventions, especially in the context of cultural factors that may hinder timely medical consultation. Given the presence of consanguinity in our patient population, genetic counseling may play a pivotal role in providing personalized reproductive advice, to help prevent genetic infertility.

## Data Availability

The raw data supporting the conclusions of this article will be made available by the authors, without undue reservation.
