# Supplementary material for: From genes to generations: genetic evaluation and counseling for infertility and pregnancy loss
Source: Front Genet. 2026 Jun 8;17:1764897. doi: 10.3389/fgene.2026.1764897 (PMC13283490; doi:10.3389/fgene.2026.1764897)
Supplement: Supplementary file 1 [file DataSheet1.pdf]

Supplementary Material

Y microdeletion testing recommendation:

Bivariate analysis demonstrated significant associations between the recommendation for Y microdeletion testing and positive semen analysis, primary infertility, and male factor infertility (p<0.001 for all), with moderate to large effect sizes. Positive semen analysis showed a particularly strong association (odds ratio (OR) 42.7, φ=0.54). Borderline associations were observed for positive reproductive-related diseases in females and positive karyotype findings. No significant association was found with family history or consanguinity (Table S1).

Table S1. Bivariate association between Y microdeletion testing recommendation and categorical predictors

Predictor	Y microdeletion testing % (by group)	Test	*p*-value	OR (95% CI)	Effect size
Consanguinity (No vs. Yes)	26.7% vs. 13.6%	Fisher	0.100	0.43 (0.17–1.11)	φ=0.13 (small)
Family history (Yes vs. No)	27.8% vs. 22.9%	Fisher	0.769	1.29 (0.43–3.87)	φ=0.04 (negligible)
Positive semen analysis (Yes vs. No)	48.1% vs. 2.1%	Fisher	<0.001	42.7 (9.85–185.24)	φ=0.54 (large)
Positive reproductive-related diseases in females (Yes vs. No)	12.2% vs. 26.9%	Fisher	0.059	0.38 (0.14–1.04)	φ=0.15 (small)
Primary vs. secondary infertility	42.5% vs. 7.4%	Fisher	<0.001	0.11 (0.04–0.26)	φ=0.41 (moderate–large)
Male/female/both infertility	Male: 56.6%<br>Female: 0.0%<br>Both: 11.0%	χ^2	<0.001	—	V=0.45 (large)
Positive karyotyping (No vs. Yes)	35.9% vs. 18.3%	Fisher	0.063	0.40 (0.17–0.97)	φ=0.20 (small–moderate)

CI: confidence interval; OR: odds ratio.

In univariate analyses of continuous predictors, female age and marriage duration were significantly associated with Y microdeletion testing recommendation. Male partners with Y microdeletion testing recommendations had partners of significantly younger age compared with those to which it was not recommended (30.0 ± 5.2 vs. 33.2 ± 5.8 years; p=0.012), with a moderate effect size (Cohen's d=0.55). Similarly, marriage duration was significantly shorter among couples with Y microdeletion testing recommendation (5.9 ± 4.7 vs. 8.4 ± 4.9 years; p=0.015), also demonstrating a moderate effect size (Cohen's d=0.52). In contrast, male age did not differ

significantly between groups (34.2 ± 5.4 vs. 35.8 ± 6.2 years; p=0.208), with only a small effect size (Cohen's d=0.27) (Table S2).

Table S2. Continuous predictors associated with Y microdeletion testing recommendation

Predictor	Y microdeletion testing recommendation (Yes vs. No)	Test	p-value	Effect size
Age of male partner (years)	34.2 ± 5.4 vs. 35.8 ± 6.2	t-test	0.208	d=0.27 (small)
Age of female partner (years)	30.0 ± 5.2 vs. 33.2 ± 5.8	t-test	0.012	d=0.55 (moderate)
Marriage duration (years)	5.9 ± 4.7 vs. 8.4 ± 4.9	t-test	0.015	d=0.52 (moderate)

The number of early pregnancy losses differed significantly according to the Y microdeletion testing recommendation status. Couples with a Y microdeletion testing recommendation had significantly fewer early pregnancy losses compared with those without it (Mann–Whitney U=1426.5, Z=−5.01, p<0.001), with a moderate effect size (r=0.38).

In multivariable logistic regression analysis, positive semen analysis findings remained the only independent predictor of Y microdeletion testing recommendation (adjusted odds ratio (aOR) 12.55, 95% CI 1.39–113.63; p=0.024). Female partner age (aOR 0.93 per year, 95% CI 0.80–1.07; p=0.301), marriage duration (aOR 0.98 per year, 95% CI 0.84–1.15; p=0.825), and infertility history variables were not independently associated after adjustment. The model demonstrated good discrimination and calibration (Nagelkerke R^2=0.384; Hosmer–Lemeshow p=0.812), indicating that semen analysis abnormalities represent the dominant independent clinical predictor for Y microdeletion testing recommendation.

Karyotyping recommendation:
Bivariate analyses of karyotyping recommendation revealed no statistically significant associations across evaluated predictors, with consistently small or negligible effect sizes. Neither parental age (male age p=0.188, d=0.26; female age p=0.144, d=0.25), marriage duration (p=0.118, d=0.32), nor the number of early pregnancy losses (Mann–Whitney U=3247.5, Z=−1.02, p=0.310, r=0.08) showed meaningful associations. Similarly, categorical factors including consanguinity, infertility characteristics, and prior karyotyping results were not significant in bivariate analyses (Tables S3–S4).

In multivariable logistic regression analysis, no demographic, clinical or reproductive history variables were independently associated with karyotyping recommendation. Positive karyotype findings (aOR 1.94, 95% CI 0.49–7.74; p=0.348), infertility etiology (overall p=0.251), number of early pregnancy losses (aOR 1.22, 95% CI 0.88–1.68; p=0.235), family history of infertility or recurrent pregnancy loss (RPL) (aOR 0.67, 95% CI 0.07–6.34; p=0.726), and consanguinity (aOR 0.33, 95% CI 0.04–2.78; p=0.308) were not independently predictive. The model demonstrated good calibration (Hosmer–Lemeshow p=0.911) but low explanatory power

(Nagelkerke R^2=0.119), indicating that karyotype recommendation is not systematically influenced by measured patient-level factors.

Table S3. Association between etiological factors and karyotype recommendation

Predictor	Karyotype recommendation % (by group)	Test	*p*-value	OR (95% CI)	Effect size
Consanguinity (No vs. Yes)	37.4% vs. 34.1%	χ^2 / Fisher	0.693	0.87 (0.42–1.77)	φ=0.03 (negligible)
Family history (Yes vs. No)	38.9% vs. 36.3%	χ^2 / Fisher	0.829	1.12 (0.41–3.04)	φ=0.02 (negligible)
Positive semen analysis (Yes vs. No)	30.6% vs. 40.8%	χ^2 / Fisher	0.639	0.85 (0.46–1.58)	φ=0.04 (small)
Positive reproductive-related diseases in females (Yes vs. No)	41.5% vs. 35.1%	χ^2 / Fisher	0.457	1.31 (0.64–2.68)	φ=0.06 (small)
Primary vs. secondary infertility	33.8% vs. 38.9%	χ^2 / Fisher	0.477	1.25 (0.67–2.33)	φ=0.05 (small)
Male/female/both infertility	Male: 35.8%<br>Female: 26.3%<br>Both: 35.2%<br>Unknown: 66.7%	χ^2	0.131	—	V=0.08 (small)
Positive karyotype (No vs. Yes)	12.8% vs. 15.5%	χ^2 / Fisher	0.704	1.25 (0.40–3.89)	φ=0.04 (negligible)

CI: confidence interval; OR: odds ratio.

Table S4. Continuous predictors associated with karyotype recommendation

Predictor	Karyotype recommendation (Yes vs. No)	Test	*p*-value	Effect size
Age of male partner (years)	34.4 ± 6.4 vs. 35.9 ± 5.9	*t*-test	0.188	d=0.26 (small)
Age of female partner (years)	31.7 ± 6.1 vs. 33.1 ± 5.6	*t*-test	0.144	d=0.25 (small)
Marriage duration (years)	6.61 ± 4.75 vs. 8.20 ± 4.95	*t*-test	0.118	d=0.32 (small)

Suspected etiology category:

Initial multinomial logistic regression analyses revealed substantial sparsity in the outcome distribution, particularly in the “undetermined/unknown” etiology category, which contained fewer than 10 observations. This resulted in multiple zero frequency cells, unstable parameter

estimates, and warnings indicating that the dependent variable had only one value observed in several subpopulations.

Therefore, the “undetermined/unknown” and the anatomical etiology category were excluded, reducing the sample size from 175 to 164 couples. Given the sample size and the multinomial nature of the outcome, a stepwise analytical strategy was adopted. Potential predictors were first screened using univariate analyses to identify variables with significant associations or meaningful effect sizes before inclusion in a multivariable multinomial logistic regression model.

Univariate multinomial regression was applied for continuous explanatory variables (parental age, marriage duration, and number of pregnancy losses), while chi-square tests were used for categorical variables (e.g., positive karyotype findings, type of infertility) (Tables S5–S6).

Table S5. Univariate associations between categorical predictors and etiology

Predictor	χ^2 (df)	*p*-value	Effect size (Cramer’s V)
Consanguinity	10.83 (2)	0.004*	0.26
Positive semen analysis	7.41 (2)	0.025*	0.21
Positive reproductive-related diseases in females	8.50 (2)	0.014*	0.21
Primary vs. secondary infertility	10.08 (2)	0.006*	0.23
Positive karyotype findings	10.13 (2)	0.006*	0.25
IVF attempted	7.13 (2)	0.028*	0.21
Family history of infertility	1.74 (2)	0.419	<0.1
Male/female infertility type	18.98 (6)	0.004*	0.24

IVF: in vitro fertilization.

Table S6. Univariate multinomial regression for continuous predictors

Predictor	Model χ^2 (df)	*p*-value	Nagelkerke R^2	aOR (95% CI)
Female age	17.51 (2)	<0.001*	0.136	0.83 (0.74–0.93)*
Male age	5.15 (2)	0.076*	0.053	0.91 (0.82–0.99)*
Marriage duration	0.28 (2)	0.869	0.003	0.99 (0.90–1.09)
Number of early pregnancy losses	1.52 (2)	0.467	0.011	1.16 (0.92–1.47)

aOR: adjusted odds ratio; CI: confidence interval.

Based on the univariate analysis, 5 predictors were chosen to ensure adequate statistical power and prevent model overfitting, considering confounder control and the sample size. The selected predictors were: (1) female age, (2) consanguinity, (3) positive karyotype findings, (4) positive semen analysis findings, and (5) primary vs. secondary infertility.

The multinomial logistic regression model was built to identify independent predictors. A total of 164 cases was available for analysis, of which 88 complete cases were included in the multinomial regression, while 76 cases were excluded due to missing data. The final multinomial model did not demonstrate a statistically significant improvement over the intercept-only model (Likelihood Ratio χ^2=15.03, df=10, p=0.131). Pseudo-R^2 measures indicated that the model explained a modest proportion of variance, with Cox & Snell R^2=0.157, Nagelkerke R^2=0.198, and McFadden R^2=0.109. Model interpretation was limited by sparse data in some outcome categories, particularly the monogenic group, which resulted in unstable parameter estimates; therefore, overall likelihood-ratio testing rather than category-specific OR was used to assess predictor contributions (Table S7).

Table S7. Multinomial (nominal) regression analysis for etiology (reference: multiple etiology group)

Predictor	χ^2 (df=2)	*p*-value	aOR (95% CI)*	Effect Size (Cohen's *w*)
Age of female partner	4.719	0.094	1.03 (0.94–1.12)	0.23
Consanguinity	2.465	0.292	1.49 (0.50–4.48)	0.17
Positive karyotype findings	6.575	0.037	0.31 (0.10–0.92)	0.27
Positive semen analysis	1.170	0.557	1.65 (0.65–4.20)	0.12
Primary vs. secondary infertility	0.756	0.685	0.77 (0.30–1.95)	0.09

aOR: adjusted odds ratio; CI: confidence interval.

To overcome the instability observed in the multinomial model due to sparse outcome categories, etiological classifications collapsed into "single etiology" and "multiple etiology," and a multivariable binary logistic regression was performed to identify independent predictors of "multiple etiology." Of 164 available cases, 88 complete cases were included in the analysis, while 76 cases were excluded due to missing data. The final model did not show a statistically significant improvement over the intercept-only model (Likelihood Ratio χ^2=8.75, df=5, p=0.120), but demonstrated acceptable calibration (Hosmer–Lemeshow p=0.066) and modest explanatory power, accounting for 9.5–12.6% of the variance according to Cox & Snell and Nagelkerke pseudo-R^2 measures.

In the multivariable model (n=88), positive karyotype findings was independently associated with the etiology, with significantly lower odds of multiple etiology (aOR 0.29, 95% CI 0.10–0.87; p=0.027), corresponding to a moderate effect size (Cohen's d=−0.67). Other predictors, including female partner age, consanguinity, abnormal semen analysis findings, and primary vs. secondary infertility, were not independently associated after adjustment and demonstrated small or negligible effect sizes. These findings indicate that positive karyotype findings are significantly associated with the single etiology category, independent of demographic and reproductive history factors (Table S8).

Table S8. Predictor effects in binary logistic regression for etiology (single vs. multiple)

Predictor	Wald χ^2	*p*-value	aOR	95% CI for aOR	Effect Size (Cohen's *d*)
Age of female partner (per year)	0.16	0.694	0.98	0.90–1.07	−0.04
Consanguinity (Yes)	0.68	0.409	1.59	0.53–4.75	0.29
Positive semen analysis (Yes)	1.04	0.308	1.62	0.64–4.07	0.31
Positive karyotype findings (Yes)	4.86	0.027	0.29	0.10–0.87	−0.67
Primary vs. secondary infertility	0.28	0.597	0.78	0.31–1.96	−0.14

aOR: adjusted odds ratio; CI: confidence interval.

Binary logistic regression was attempted to identify predictors of outcome recommendations. However, due to the small number of cases and substantial missing data, the model showed separation and unstable estimates, so stepwise approach was used to identify the predictors to be included in the multivariant binary logistic regression.

IVF with PGT-A recommendation:
In vitro fertilization with preimplantation genetic testing for aneuploidies (IVF with PGT-A) recommendation demonstrated a distinct bivariate pattern consistent with aneuploidy-risk–driven clinical decision-making. Female partner age was significantly higher among couples receiving a recommendation for PGT-A (p=0.002), with a moderate effect size (d=0.55). Positive karyotype findings showed a strong association with PGT-A recommendation (p<0.001), with a large effect size (φ=0.49) and substantially increased odds (OR 9.76, 95% CI 3.85–24.72). Infertility etiology (male/female) was also significantly associated with it (p=0.009; Cramer's V=0.26), indicating differential use of PGT-A across infertility types. Marriage duration showed a borderline association (p=0.059; d=0.39), the number of early pregnancy losses did not differ significantly between couples who were recommended to have IVF with PGT-A and those who were not (Mann–Whitney U=3630.5, Z=−0.20, p=0.841), with a negligible effect size (r=0.02). These results support a multivariable model emphasizing maternal age, chromosomal findings, and infertility etiology, while excluding early pregnancy loss burden as a driver of PGT-A use.

In multivariable logistic regression analysis, female partner age, karyotype status, and infertility etiology emerged as independent predictors of IVF with PGT-A recommendation. Each one-year increase in female age was associated with higher odds of undergoing PGT-A (aOR 1.62, 95% CI 1.04–2.54; p=0.034). The presence of a positive karyotype finding was the strongest independent predictor of a PGT-A recommendation (aOR 342.71, 95% CI 5.78–20,320.37; p=0.005). Infertility etiology (male/female) was also independently associated with a PGT-A recommendation (overall p=0.014), with both female factor infertility (aOR 175.18, 95% CI 2.48–12,389.59; p=0.017) and combined infertility (aOR 137.65, 95% CI 4.46–4,251.84; p=0.005) showing significantly higher odds compared with male factor infertility. Marriage duration showed a borderline inverse association (p=0.064), while primary versus secondary infertility was not independently associated with it (p=0.177). The model demonstrated excellent discrimination and calibration (Nagelkerke R^2=0.726; Hosmer–Lemeshow p=0.545).

Table S9. Association between etiological factors and IVF with PGT-A

Predictor	IVF with PGT-A % (by group)	Test	*p*-value	OR (95% CI)	Effect size
Consanguinity (No vs. Yes)	61.1% vs. 54.5%	χ² / Fisher	0.446	0.77 (0.38–1.53)	φ=0.06 (negligible)
Family history (Yes vs. No)	38.9% vs. 61.8%	χ² / Fisher	0.061	0.39 (0.15–1.07)	φ=0.14 (small)
Positive semen analysis (Yes vs. No)	54.3% vs. 63.8%	χ² / Fisher	0.220	0.67 (0.37–1.23)	φ=0.01 (negligible)
Positive reproductive-related diseases in females (Yes vs. No)	65.9% vs. 57.5%	χ² / Fisher	0.338	1.43 (0.69–2.97)	φ=0.07 (small)
Primary vs. secondary infertility	55.0% vs. 63.2%	χ² / Fisher	0.274	1.40 (0.77–2.57)	φ=0.08 (small)
Male/female/both infertility	Male: 41.5% Female: 78.9% Both: 65.9%	χ²	0.009	—	V=0.26 (moderate)
Positive karyotype findings (No vs. Yes)	38.5% vs. 85.9%	χ² / Fisher	<0.001	9.76 (3.85–24.72)	φ=0.49 (large)

CI: confidence interval; IVF with PGT-A: in vitro fertilization with preimplantation genetic testing for aneuploidies; OR: odds ratio.

Table S10. Continuous predictors associated with IVF with PGT-A

Predictor	IVF with PGT-A (Yes vs. No)	Test	*p*-value	Effect size
Age of male partner (years)	36.0 ± 5.2 vs. 34.7 ± 7.0	*t*-test	0.261	d=0.21 (small)
Age of female partner (years)	33.8 ± 5.8 vs. 30.7 ± 5.5	*t*-test	0.002	d=0.55 (moderate)
Marriage duration (years)	8.35 ± 4.78 vs. 6.47 ± 5.00	*t*-test	0.059	d=0.39 (small–moderate)

IVF with PGT-A: in vitro fertilization with preimplantation genetic testing for aneuploidies.

IVF with PGT-M recommendation:

Bivariate analysis demonstrated that consanguinity and the presence of a positive reproductive-related disease in females were significantly associated with increased recommendation of IVF with preimplantation genetic testing for monogenic disorders (PGT-M) (p=0.022 and p=0.030, respectively). Positive semen analysis findings were inversely associated with an IVF with PGT-M recommendation (p<0.002). Additionally, infertility etiology (male, female, both

or unknown) showed a significant association with a PGT-M recommendation ($\chi^2 = 18.21$, $p < 0.001$). No significant associations were observed with family history, primary versus secondary infertility or karyotype findings (Table S11).

Table S11. Bivariate association with IVF with PGT-M

Predictor	IVF with PGT-M % by group	Test	*p*-value	OR (95% CI)	Effect size
Consanguinity (No vs. Yes)	24.4% vs. 43.2%	Fisher	0.022*	2.35 (1.15–4.82)	φ=0.18 (small–moderate)
Family history (Yes vs. No)	22.2% vs. 29.9%	Fisher	0.593	0.67 (0.21–2.14)	φ=0.05 (negligible)
Positive semen analysis (Yes vs. No)	17.3% vs. 39.4%	Fisher	0.002*	0.322 (0.13–0.60)	φ=0.24 (moderate)
Positive reproductive-related diseases in females (Yes vs. No)	43.9% vs. 24.6%	Fisher	0.030*	2.40 (1.15–4.98)	φ=0.18 (small–moderate)
Primary vs. secondary infertility	23.8% vs. 33.7%	Fisher	0.182	1.63 (0.84–3.18)	φ=0.11 (small)
Male/female/both infertility	Male: 11.3%<br>Female: 42.1%<br>Both: 31.9%<br>Unknown: 66.7%	χ^2	<0.001*	—	V=0.27 (moderate)
Positive karyotype findings (No vs. Yes)	23.1% vs. 16.9%	Fisher	0.455	0.68 (0.26–1.79)	φ=0.08 (small)

CI: confidence interval; IVF with PGT-M: in vitro fertilization with preimplantation genetic testing for monogenic disorders; OR: odds ratio.

In univariate analyses of continuous predictors, marriage duration was significantly associated with a recommendation of IVF with PGT-M. Couples recommended to undergo IVF with PGT-M had a significantly longer marriage duration compared with those who were not (9.36 ± 5.82 vs. 7.12 ± 4.51 years; *p*=0.046), with a moderate effect size (Cohen's d=0.46). In contrast, neither male age (33.9 ± 5.9 vs. 35.9 ± 6.1 years; *p*=0.116; Cohen's d=0.33) nor female age (32.9 ± 6.1 vs. 32.5 ± 5.7 years; *p*=0.690; Cohen's d=0.07) differed significantly between groups (Table S12).

Table S12. Continuous predictors associated with IVF with PGT-M

Predictor	IVF with PGT-M (Yes vs. No)	Test	*p*-value	Effect size
Age of male partner (years)	33.9 ± 5.9 vs. 35.9 ± 6.1	*t*-test	0.116	d=0.33 (small–moderate)
Age of female partner (years)	32.9 ± 6.1 vs. 32.5 ± 5.7	*t*-test	0.690	d=0.07 (negligible)

Marriage duration (years)	9.36 ± 5.82 vs. 7.12 ± 4.51	*t*-test	0.046*	d=0.46 (moderate)

IVF with PGT-M: in vitro fertilization with preimplantation genetic testing for monogenic disorders.

The number of early pregnancy losses was significantly higher among couples recommended to undergo IVF with PGT-M compared with those who were not (Mann–Whitney U=2544.5, Z=−2.18, p=0.029), with a small effect size (r=0.16). These findings justified a multivariable model where we included marriage duration, abnormal semen analysis, consanguinity, positive reproductive-related diseases in females, and infertility etiology (male/female/both) in the multivariable analysis.

In multivariable logistic regression, none of the variables retained independent statistical significance for predicting a recommendation of IVF with PGT-M. Marriage duration was no longer independently associated (aOR 1.06 per year, 95% CI 0.96–1.18; p=0.259), nor were abnormal semen analysis (aOR 0.43, 95% CI 0.14–1.34; p=0.145), consanguinity (aOR 2.19, 95% CI 0.74–6.47; p=0.156) or reproductive medical history in females (aOR 2.04, 95% CI 0.72–5.77; p=0.177). Infertility etiology did not demonstrate an independent association after adjustment (overall p=0.703).

The model showed excellent calibration (Hosmer–Lemeshow p=0.997) but modest explanatory power (Nagelkerke R^2=0.223), indicating that a PGT-M recommendation is influenced by multifactorial clinical considerations rather than any single independent predictor.
